# Editorial: Managing metal toxicity in plants and soil: strategies for stress mitigation and remediation

**DOI:** 10.3389/fpls.2026.1827865

**Published:** 2026-04-20

**Authors:** Muhammad Shoaib Rana, Yousif Abdelrahman Yousif Abdellah, Aamir Hamid Khan, Lin Hu, Ruilong Wang

**Affiliations:** 1 State Key Laboratory of Ecosystem Safety and Sustainable Development in Arid Lands, Xinjiang Institute of Ecology and Geography, Chinese Academy of Sciences, Urumqi, Xinjiang, China; 2 Fukang Station of Desert Ecology, Chinese Academy of Sciences, Fukang, Xinjiang, China; 3 The Germplasm Bank of Wild Species, Yunnan Key Laboratory for Fungal Diversity and Green Development, Kunming Institute of Botany, Chinese Academy of Sciences, Kunming, Yunnan, China; 4 Department of Biogeography, Paleoecology and Nature Conservation, Faculty of Biology and Environmental Protection, University of Łódź, Łódź, Poland; 5 Nanning Normal University, Nanning, Guangxi, China; 6 College of Natural Resources and Environment, South China Agricultural University, Guangzhou, China

**Keywords:** crop stress physiology, heavy metal stress, metal detoxification, molecular responses, phytoremediation, soil-plant amendments

Heavy metal and metalloid contamination in soils has emerged as one of the most persistent environmental challenges affecting agricultural productivity, ecosystem stability, and food security worldwide (Rana et al., 2025; Sweta and Singh, 2024; Hou et al., 2020). Anthropogenic activities such as mining, industrial discharge, excessive agrochemical use, and urbanization have significantly increased the accumulation of toxic metals and metalloids in terrestrial ecosystems. These contaminants disrupt plant physiological processes, impair soil microbial communities, and pose substantial risks to human health through the food chain (Angon et al., 2024). At the same time, certain micronutrients such as iron (Fe), manganese (Mn), and boron (B), which are essential for plant growth at low concentrations, can become toxic when present in excess. Understanding the mechanisms underlying plant tolerance to metal stress and developing sustainable strategies for soil remediation therefore represent critical priorities for modern agriculture and environmental management *(*Mohamed et al., 2025).

The Research Topic “Managing Metal Toxicity in Plants and Soil: Strategies for Stress Mitigation and Remediation” brings together diverse studies addressing the physiological, molecular, ecological, and technological aspects of metal stress management in plants and soils. The articles included in this Research Topic highlight recent advances in plant stress physiology, genetic regulation, soil amendment strategies, nanotechnology-based interventions, and phytoremediation approaches aimed at mitigating heavy metal toxicity and restoring ecosystem functionality.

A central theme across several contributions is the elucidation of plant physiological and biochemical responses to metal stress. For example, *Pan* et al. investigated how planting density and nitrogen application influence the tolerance of *Xanthium strumarium* subsp. *sibiricum* under manganese stress. Their findings demonstrate that optimized nitrogen supply and planting density can enhance antioxidant enzyme activities, promote osmotic adjustment, and reduce manganese accumulation in plant tissues, thereby improving plant resilience under metal stress conditions. In a complementary study, *Chalchisa* et al. explored the physiological and molecular responses of kiwifruit species to excessive boron exposure. Their study revealed significant declines in chlorophyll content, biomass production, and stress-related gene expression under elevated boron levels, providing valuable insights into the mechanisms governing boron toxicity and tolerance in horticultural crops.

At the molecular level, understanding the genetic regulation of metal transport and detoxification is essential for developing stress-resilient crop varieties. *Zhang* et al. conducted a comprehensive genomic characterization of the metal tolerance protein (MTP) gene family in *Coptis chinensis*. Their work identified 25 MTP genes and highlighted specific members that likely contribute to heavy metal tolerance by mediating metal ion sequestration and compartmentalization, particularly through vacuolar transport, thereby helping reduce metal toxicity and maintain cellular homeostasis. Likewise, *Mesci and Çatal* investigated the expression of stress-related genes including ascorbate peroxidase (APX), catalase (CAT), metallothionein (MT), and phytochelatin synthase (PCS) in forage pea (*Pisum sativum* ssp. *arvense*) exposed to nickel and lead stress. Their results showed differential transcriptional responses depending on cultivar and treatment level, with marked upregulation in APX, CAT, MT and PCS gene expressions were detected mostly at high nickel sulphate and lead acetate trihydrate applied rates., providing molecular evidence for adaptive defense mechanisms against toxicity. Another key research direction represented in this Research Topic concerns genetic improvement and crop breeding strategies aimed at balancing metal tolerance with nutritional quality. *Sonu* et al. applied genome-wide association analysis to identify genetic loci controlling grain iron and zinc concentrations in rice grown under contrasting soil iron conditions. Their study identified several significant marker-trait associations and candidate genes involved in metal homeostasis, offering valuable resources for biofortification breeding programs that seek to enhance micronutrient content while maintaining tolerance to excess soil metals.

Beyond plant genetics and physiology, several contributions emphasize soil based and technological strategies for mitigating metal contamination. *Iqbal* et al. demonstrated that vermicompost application significantly improved soil fertility, enhanced nitrogen assimilation, and reduced cadmium accumulation in fragrant rice. By improving soil organic carbon and nutrient availability, vermicompost also stimulated physiological activities and grain quality, highlighting its potential as an environmentally friendly amendment for contaminated soils. Similarly, *Jiang* et al. reported that biochar co-compost effectively alleviated lead toxicity in *Brassica napus* by improving soil properties, enhancing antioxidant defense systems, and reducing metal uptake. Complementing these findings, *Sheng* et al. showed that biochar addition increased the efficiency of rapeseed-based phytoremediation in copper contaminated soils while simultaneously improving plant biomass and yield. Together, these studies demonstrate the promising role of organic amendments in stabilizing heavy metals, improving soil health, and enhancing crop productivity in polluted environments.

Phytoremediation, which utilizes plants to extract, stabilize, or transform contaminants, represents another sustainable strategy for managing metal-polluted ecosystems. *Sirgedaitė-Šežienė* et al. evaluated the phytoremediation potential of *Populus tremula* L. and *Salix caprea* L. under arsenic, cadmium, and lead stress. Their findings revealed species-specific differences in metal uptake, antioxidant responses, and nutrient homeostasis, with *S. caprea* L. showing promising potential for cadmium phytoextraction and both species demonstrating Phyto-stabilization capacity for lead. In a field-based ecological assessment, *Deng* et al. investigated plant communities in copper mine tailings in Southwest China and identified several species, including *Salix balfouriana*, with strong metal accumulation capabilities suitable for ecological restoration of contaminated mining landscapes. Phytoremediation is increasingly strengthened through the integration of nanotechnology and biologically mediated processes, which together enhance remediation efficiency in contaminated soils. In this Research Topic, several studies highlight the important role of nanotechnology in supporting phytoremediation processes. For instance, *Liu* et al. demonstrated that humic acid-loaded nano zero-valent iron, when combined with microbial communities, enhances the removal of chromium and cadmium through a synergistic mechanism, in which microbial extracellular polymers facilitate metal complexation and reduce metal toxicity, thereby improving overall remediation efficiency. Similarly, *Tabassam* et al. reported that the application of cerium oxide nanoparticles improved plant growth, nutrient uptake, and antioxidant enzyme activities while reducing arsenic accumulation in plant tissues, indicating their potential to enhance phytoremediation efficiency under metal stress. Supporting this perspective, *Cao* et al. emphasized that nanoparticles can improve plant stress tolerance, nutrient use efficiency, and overall crop performance, thereby strengthening plant-based remediation strategies. In addition to nanotechnology, biologically mediated processes play a fundamental role in phytoremediation. Although not always directly measured, the improvements in soil properties and plant physiological responses are important in soil microbial activity in enhancing nutrient cycling, reducing metal bioavailability, and promoting plant growth under contaminated conditions. Furthermore, the study by *Sun* et al. highlights how soil biological interactions, including litter decomposition and nutrient dynamics, can influence plant-soil feedback mechanisms, which are critical for ecosystem-level responses to environmental stress. These findings demonstrate that the integration of nanotechnology and soil biological processes provides a more effective and sustainable framework for improving phytoremediation performance.

Collectively, the studies presented in this Research Topic provide a comprehensive overview of current progress in understanding and managing metal toxicity in plants and soils. They highlight the importance of integrating molecular biology, plant physiology, soil science, and environmental technology to develop effective mitigation strategies. From identifying genetic determinants of metal tolerance to exploring innovative soil amendments and nanotechnology-based solutions, the research showcased here underscores the multidimensional nature of metal stress management.

Despite these advances, several challenges remain. Future research should further explore the long-term environmental implications of remediation technologies, particularly the ecological impacts of nanomaterials and large-scale soil amendments. Integrating omics-based approaches, precision agriculture technologies, and ecological restoration strategies will also be essential for improving the sustainability and effectiveness of metal stress mitigation. In summary, this article highlights the growing body of knowledge dedicated to understanding plant responses to metal toxicity and developing sustainable remediation strategies. By combining insights from molecular mechanisms to field-scale applications, the contributions in this Research Topic advance our understanding of how plants and soils can be managed to mitigate metal stress, enhance ecosystem resilience, and support global food security.
